# Soil organic carbon storage in a mountain permafrost area of Central Asia (High Altai, Russia)

**DOI:** 10.1007/s13280-020-01433-6

**Published:** 2020-12-07

**Authors:** Didac Pascual, Peter Kuhry, Tatiana Raudina

**Affiliations:** 1 Department of Physical Geography and Ecosystem Sciences, Lund University, 223 62 Lund, Sweden; 2 Department of Physical Geography, Stockholm University, 106 91 Stockholm, Sweden; 3 Tomsk State University, 634050 Tomsk, Russia

**Keywords:** Central Asia, Climate change impacts and feedbacks, Mountain permafrost, Soil organic carbon

## Abstract

The thawing and subsequent decomposition of large stocks of soil organic carbon (SOC) currently stored in the northern circumpolar permafrost region are projected to result in a ‘positive’ feedback on global warming. The magnitude of this feedback can only be assessed with improved knowledge about the total size and geographic distribution of the permafrost SOC pool. This study investigates SOC storage in an under-sampled mountain permafrost area in the Russian High Altai. SOC stocks from 39 soil pits are upscaled using a GIS-based land cover classification. We found that the top 100 cm of soils in Aktru Valley and the adjacent Kuray Basin only holds on average 2.6 ± 0.6 kg C m^−2^ (95% confidence interval), of which only c. 1% is stored in permafrost. Global warming will result in an upward shift of alpine life zones, with new plant cover and soil development at higher elevations. As a result, this type of mountain permafrost area might act as a net C sink in the future, representing a ‘negative’ feedback on global warming.

**Electronic supplementary material:**

The online version of this article (10.1007/s13280-020-01433-6) contains supplementary material, which is available to authorized users.

## Introduction

The northern circumpolar permafrost region represents about 16% of the global soil area, stretching from the High Arctic in the north to mid-latitude mountain and continental areas in the south (Tarnocai et al. 2009). At high latitudes, temperatures have increased at a rate of 0.6 °C per decade over the last 30 years, twice as fast as the global average (IPCC 2013), and temperatures have also increased in the mountain permafrost areas to the south (e.g., Zhang et al. 2018). These trends are expected to continue during the 21st Century. Under these warming conditions, near-surface permafrost areas are expected to decrease by 37 to 81% by the end of the Century (IPCC 2013).

Permafrost has been recognized as one of the vulnerable carbon (C) pools in the Earth System (Gruber et al. 2004). The fate of permafrost C in a warming climate has received increased attention, both in the scientific community as well as the public in general. This interest was fueled by a new and high estimate of the total amount of soil organic carbon (SOC) stored in the northern circumpolar permafrost region (Tarnocai et al. 2009). These stocks are susceptible to thawing and decomposition under rising temperatures, resulting in the release of greenhouse gases to the Earth’ atmosphere and leading to even more global warming and permafrost thawing (the so-called ‘positive’ permafrost C feedback).

International research on this topic has thus increased exponentially over the last decade. The most recent review on the permafrost C feedback (Schuur et al. 2015) suggested that C release from thawing permafrost will not result in rapid and irreversible global warming, but should be carefully considered when considering international policy efforts such as the Paris COP21 agreement on keeping global temperature increases below 1.5 (2) °C (UNFCCC 2015).

To better assess the potential magnitude of the permafrost C feedback, it is crucial to constrain the total size, geographic distribution and characteristics of the permafrost SOC pool. Hugelius et al. (2014) estimated SOC stocks in the northern circumpolar permafrost region to be c. 1300 PgC (with an uncertainty range of 1100 to 1500 PgC). However, data for extensive areas of the northern circumpolar permafrost region is still scarce, particularly in the High Arctic and in regions with thin sedimentary overburden, including mountains and highlands.

Recent studies indicate that, in contrast to lowland permafrost areas, mountain areas often store relatively small amounts of SOC in permafrost (e.g., Fuchs et al. 2015; Wojcik et al. 2019), and could therefore become a net C sink under warming conditions, since the C uptake through new vegetation and soil development at higher elevations is likely to exceed the small C losses from thawing and decomposing permafrost C stocks. The objective of this study is to provide a detailed SOC inventory for an under-sampled mountain permafrost area in the Russian High Altai. The Altai mountain range in Central Asia, extending through Kazakhstan, Russia, Mongolia and China, has experienced a relatively rapid warming over recent decades (Zhang et al. 2018; Li et al. 2020), along with a substantial altitudinal treeline advancement (Gatti et al. 2019). Results are discussed in the context of a potential ‘negative’ permafrost C feedback on global warming in mountain areas. This paper contributes to a series of studies on Siberian environmental change (Callaghan et al. 2021).

## Study area

**Fig. 1:**
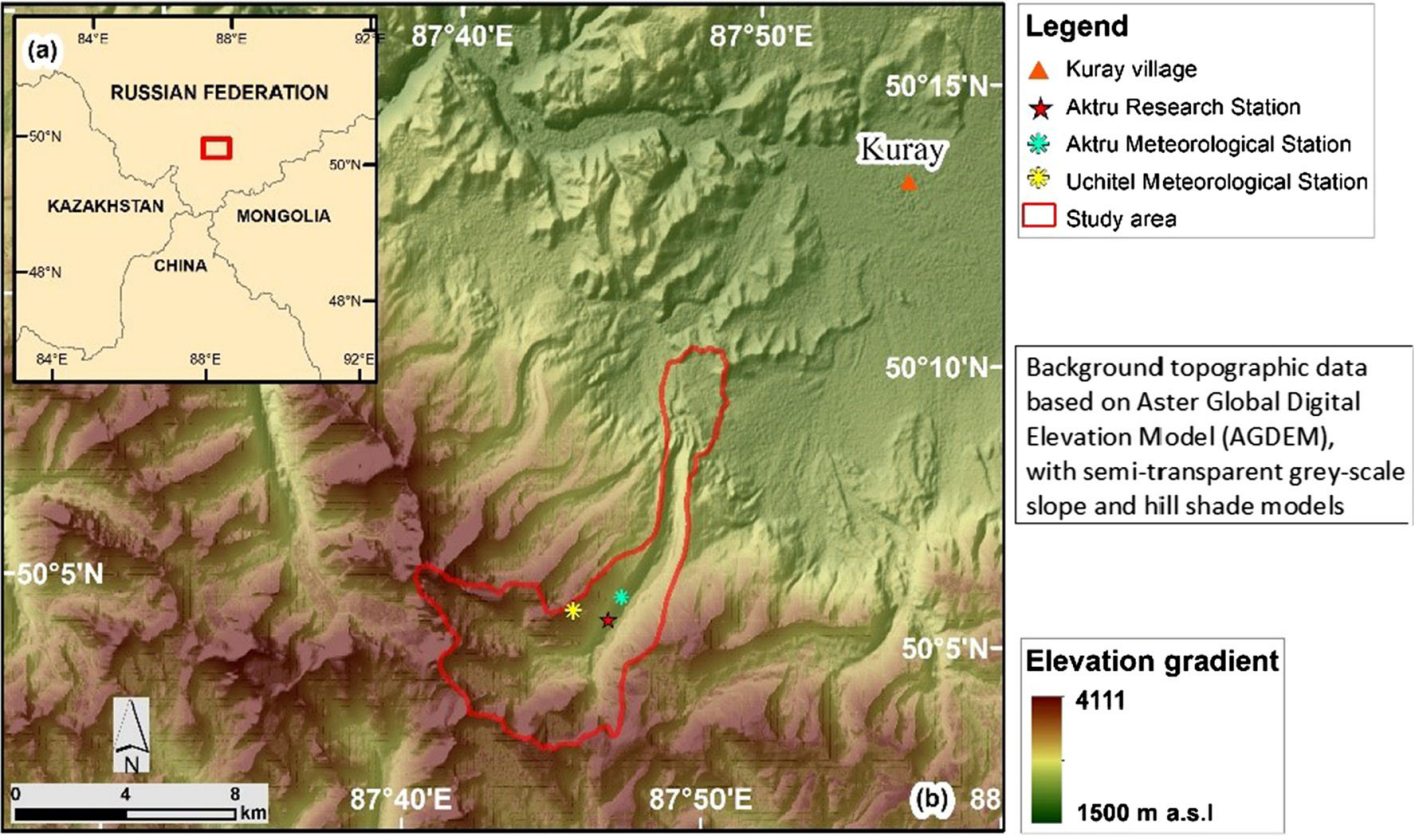
**a** Study area location in the High Altai region of Russia, with country borders; **b** Study area delimitation (red line) in Aktru Valley and the adjacent Kuray Basin, with places of interest

The study was conducted in Aktru Valley and the adjacent intra-montane Kuray Basin (50 °N, 87 °E), a mid-latitude mountainous area in the Russian High Altai (Fig. 1). The study area (50.9 km^2^) was delineated based on the catchment of Aktru River and on geomorphological landforms of Pleistocene age formed by glaciers extending from Aktru Valley into Kuray Basin. The altitudinal range of the study area extends from about 1600 to 4000 m. Bedrock in the upper part of the study area is mainly represented by sericite-chlorite slates, phyllites, quartzites and very sporadically calcites (Galakhov et al. 1987). A large glacier occupied all of Aktru Valley at the time of the Last Glacial Maximum. However, there is landform evidence of older, even more extensive glaciation in Kuray Basin (Lehmkuhl et al. 2011). Several glacier re-advances have been reported in the middle and upper part of Aktru Valley during the Late Holocene Neoglacial (Agatova et al. 2012).

The study area is characterized by strong climatic gradients. Figure 1 shows the location of two meteorological stations within the study area: Aktru station (2150 m) and Uchitel station (3050 m). Mean annual air temperature (MAAT) in Aktru station is − 5.2 °C (1971–1993), ranging from − 21.6 °C in January to 9.7 °C in July (Sevastyanova and Sevastyanov 2013). In Uchitel, the MAAT is − 9.3 °C, ranging from − 23.6 in January to 4.5 °C in July (data from winter months provided by S.V. Kharlamov, pers. comm.). The inferred altitudinal temperature lapse rate for the month of July is 0.58  °C/100 m. Precipitation varies largely across an altitudinal gradient due to the extreme topography. Mean annual precipitation in Aktru station is 520 mm (Sevastyanov 1998), while at higher elevations it is about 1000 mm (Tronov et al. 1973). The lowest part of the study area, in Kuray Basin (1600–1800 m), is characterized by a dry continental climate caused by its intra-montane location. MAAT in the town of Kosh-Agach, located c. 65 km to the ESE of the study area with a similar topographic setting to Kuray Basin, is − 4.2 °C (1981–2010). Mean annual precipitation is 122 mm (RIHMI-WDC 2018). According to Li et al. (2020), MAAT in the Altai Mountains has increased at a rate of 0.41 °C per decade over the period 1970–2015. Mean annual air temperature increases in the order of 3–5 °C are projected for Central Asia by the end of this Century (IPCC 2013).

**Fig. 2:**
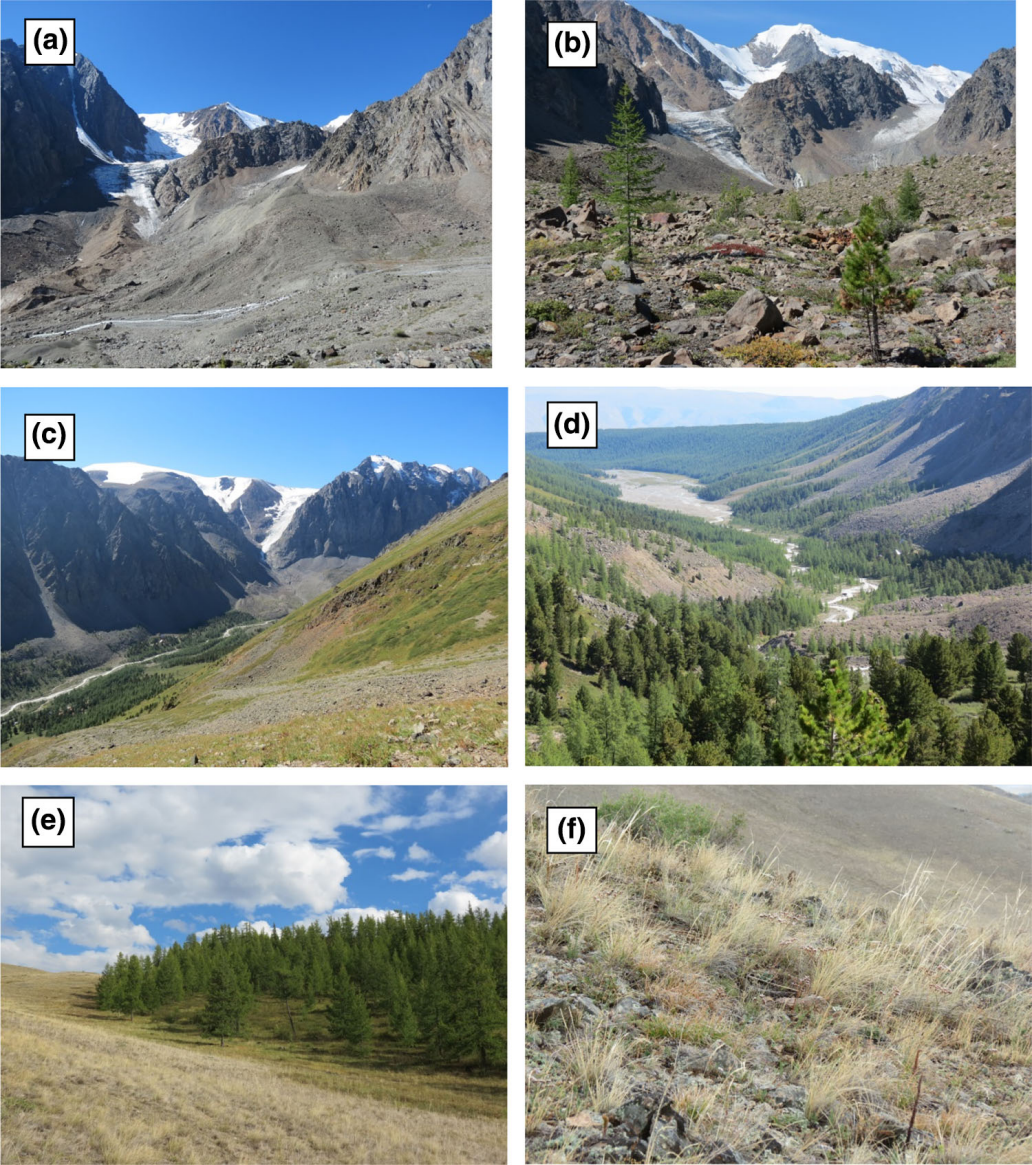
Main land cover classes in the study area: **a** bare ground on recently deglaciated terrain; **b** patchy vegetation on Little Ice Age moraine; **c** alpine tundra on mountain slope, with Aktru research/tourist stations in the valley; **d** subalpine forest with Aktru river floodplain in the background; **e** steppe forest on N facing hill slope in Kuray Basin; and, **f** steppe grassland on S facing hill slope in Kuray Basin. Photos by Peter Kuhry

The predominant vegetation cover in the study area changes along these climatic gradients. In the lowest parts (1600–1800 m), steppe grassland is characteristic with steppe forest patches mostly on N facing hill slopes. The middle part of the valley (1800 to 2300 m) is dominated by mountain and subalpine forests (mainly *Larix* and *Pinus* species). Some trees reach up to 2500 m. Nevertheless, between 2300 and 2600 m vegetation is mostly composed of alpine tundra species. Above 2600 m, vegetation becomes very sparse, with a few patches of lichens, mosses or grasses, which tend to disappear as elevation increases. On the active river floodplain, in recently deglaciated areas and on very steep slopes, vegetation is generally sparse. About 25% of the study area is covered by glaciers, but these are currently undergoing a rapid retreat (Narozhniy and Zemtsov 2011). Figure 2 shows the most important land cover types in the study area; a more detailed description of the vegetation composition in different land cover types based on plant functional types is provided in Table S1.

The study area is characterized by continuous permafrost in Aktru Valley and discontinuous permafrost in Kuray Basin (Yershov 2003). Soils are dominated by non-Gelisols and, sporadically, Orthels. Soils in Aktru Valley are generally poorly developed (< 40 cm deep), while in the adjacent Kuray Basin soil depth ranges from 10 to 30 cm on steeper hills, to > 100 cm in areas dominated by depositional processes.

## Methods

### Field sampling

A total of 39 soil profiles were described and sampled between 1600 and 2600 m of elevation (Table S1), by applying a semi-random sampling scheme along five transects (AK-T1 to AK-T5) representing the different land cover types in the study area (August 2017). Transects were 600–1500 m long, with strictly pre-defined sampling intervals of 100 or 150 m using a hand-held GPS device, resulting in 7–11 profile sites per transect. This approach eliminates bias in the exact location of sampling sites. The objective of this sampling strategy was to obtain sufficient but unbiased soil profile replication, particularly for the most important land cover types in the study area. At each site, we described plant cover, fractions of large stones and of fine-grained mineral bare ground at the surface, landform and topography (elevation, slope and aspect). High elevation areas were documented and photographed along a hiking trail, at approximately 100 m contour intervals between 2400 and 2900 m (*n* = 6).

In total, 234 soil samples were collected. Sampling at each site was (nearly) continuous along the full depth of the profile, except for deep and homogenous soil horizons, where cylinders with 6 cm in diameter could represent depth intervals of up to c. 10 cm. The topsoil organic layer (OL), where present, was sampled by cutting out and measuring the dimensions of blocks of soil material. Two additional randomly selected OL replicates were collected to acquire a more representative sample. Below the top OL, in shallow and stony soils, samples were collected using a 100-cm^3^ cylinder to as deep as bedrock or stones allowed. In deep and fine-grained soils, as well as in the permafrost layer (where reached), samples were retrieved every c. 10 cm by incrementally hammering a steel pipe (Ø = 4.2 cm) into the ground. These approaches ensured that known field volumes of soil material were collected, which after drying could be used to calculate dry bulk densities. Sampling was conducted until the reference depth of 100 cm was reached or bedrock/large stones were hit. Volume of large stones (> 4 cm) was visually estimated for the surface (radius of 5 m around the profile site) and for each sampled depth interval (CF_large_, volume %). For every sample, additional metadata was collected including exact depth interval, occurrence of roots, occurrence of permafrost and buried C-enriched soil pockets (if applicable).

Another 10 samples of stones were taken to estimate the dry bulk density (DBD, g cm^−3^) of the coarse fraction following Rytter (2012). In addition, nine samples of large woody roots were taken to estimate their DBD based on dry weight (after oven drying them at 65 °C for 4 days) and volume (estimated by water displacement). Additional observations on root penetration into coarse colluvium, till and glacio-fluvial materials underlying the soil profiles were made at uprooted trees and natural exposures in the study area. Even though sampling was conducted before maximum annual thaw is normally reached, unfrozen and frozen soil horizons are referred to as active layer and permafrost layer to facilitate the discussion. Most soil profiles did not present well defined soil genetic horizons below the OL.

### Soil chemical analysis and radiocarbon dating

Soil samples (*n* = 234) were weighed in the laboratory before and after oven drying at 65 °C for 4 days to calculate their gravimetric water content (%) and DBD. An additional test on a subset of samples showed negligible further weight losses after oven drying at 105 °C. Subsequently, all samples with a coarse fraction were sieved through a 2 mm mesh and all stones (≥ 2 mm) were weighed to measure the small stone fragment fraction in the sample (CF_small_, weight %). For samples collected from 14 soil profiles in Aktru Valley and the adjacent Kuray Basin (selected to represent all land cover classes in the study area) the weight of roots on the sieve was measured to calculate the contribution of root material to total SOC storage. These roots (and other coarse organic material) were subsequently returned to the remaining fine fraction sample before further analysis. Loss on ignition (LOI, weight %) at 550 °C (for 2 h) was used to estimate the soil organic matter (SOM) content, and at 950 °C (for 2 h) to estimate the inorganic carbon (IC) content in each sample. For a few unsampled depths, soil properties were interpolated from adjacent samples, considering any relevant field observations such as soil texture, percentage of stones, root occurrence, etc.

A total of 41 samples from eight profiles, selected to represent the main vegetated land cover classes in the study area, were processed in a Carlo Erba Elemental Analyzer NC2500 attached to a Finnigan MAT Delta Plus mass spectrometer to determine their elemental C (%C) and total nitrogen content as well as their δ^13^C isotopic signals. A subset of 12 of these 41 soil samples (representing two profiles with high carbonate content, as suggested by the high LOI at 950 °C, high C/N ratios and less depleted δ^13^C values in their mineral subsoil samples), were re-sampled, acid-treated with HCl_2_, and re-analyzed. These analyses allowed us to establish the C content, C/N ratio and δ^13^C signature of organic matter in profiles affected by carbonate enrichment. Third-order polynomial regressions between %C and organic matter (OM) were used to calculate the %C content for the remaining samples. Equation (1) (*n* = 29, *R*^2^ = 0.90) was applied to soil profiles with low carbonate content, whereas Eq. (2) (*n* = 12, *R*^2^ = 0.99) was applied to profiles with high carbonate content.

1 $$\% {\text{C }} = \, - 0.0000 8 9 { }* \, \left( {\text{OM}} \right)^{ 3} + \, 0.0 1 1 6 4 6 { }* \, \left( {\text{OM}} \right)^{ 2} + \, 0. 1 7 1 3 8 5 { }* \, \left( {\text{OM}} \right)$$

2 $$\% {\text{C }} = \, - 0.0000 4 4* \, \left( {\text{OM}} \right)^{ 3} + \, 0.00 8 5 6 6 { }* \, \left( {\text{OM}} \right)^{ 2} + \, 0. 1 3 6 8 8 7 { }* \, \left( {\text{OM}} \right)$$

The organic carbon/nitrogen (OC/N) ratios and δ^13^C values of samples are useful indicators of SOM decomposition (e.g., Kuhry and Vitt 1996; Ping et al. 2015), and can be used to assess the burial processes of deeper C-enriched soil pockets (e.g., Palmtag et al. 2015).

The C content of large woody roots was also determined by Elemental Analyzer (49.7% ± 1.57; *n* = 9).

Six samples were submitted for AMS ^14^C dating to the Radiocarbon Laboratory in Poznan, Poland. The resulting ages were calibrated to calendar years, cal year BP (1950), using OxCal 4.3 (Bronk Ramsey 2019).

### SOC storage calculations

The SOC content (kg C m^−2^) for each sample was calculated using Eq. (3), where *T* is the depth interval in cm, %C is the weight fraction of organic carbon in the soil, *DBD* (g cm^−3^) is the dry bulk density of the sample, CF_large_ is the volume fraction of large stones described from the soil pits (CF > 4 cm), CF_small_ is the weight fraction of the small stones in the sample (CF > 2 mm), and 10 is for unit conversion:

3 $${\text{SOCC}} = \, \left( {1 \, {-}{\text{ CF}}_{\text{large}} } \right) \, * \, C \, *{\text{ DBD }}* \, \left( {1 \, {-}{\text{ CF}}_{\text{small}} } \right) \, * \, T \, * \, 10$$

SOC storage in each soil profile was calculated for the standard depth interval 0–100 cm, although in many cases the depth of 100 cm was not possible to reach due to the presence of large stones or bedrock. The interval between the depth at which sampling was stopped due to stones and 100 cm was assumed to contain negligible SOC. To further characterize vertical partitioning, SOC storage was calculated separately for the topsoil organic layer and the permafrost layer.

SOC calculations were performed for the entire study area and its vegetated fraction, since SOC storage is largely expected under vegetated areas only. Especially in high-alpine terrain, but also on floodplains, plant cover is very sparse. In these cases, SOC storage obtained for vegetated patches was only applied to the fraction with plant cover, whereas for the fraction of adjacent bare ground SOC content was obtained using only values from mineral subsoil samples.

The contribution of roots to SOC storage was calculated using Eq. (3) and sample root dry weights multiplied by a factor of 0.497 (representing average C content of root material) obtained for 14 selected soil profiles representing all vegetated land cover classes in the study area.

### Land cover upscaling

A land cover classification (LCC) for the 50.9 km^2^ study area was derived from Landsat 8 satellite images with pixel size 30 m * 30 m (Earth Explorer; 14 August 2017). A maximum likelihood supervised classification was applied using the spectral bands 2–5 (Campbell 2011). The training polygons for the different land cover classes were manually defined using ArcGis 10.5, based on visual interpretation of the Landsat 8 image. Due to the relatively low spatial resolution of the available satellite imagery, it was not possible to spectrally separate very small areas of the land cover class “shrub wetland”, which is dominated by c. 50 cm tall *Betula* shrubs and has exceptional relevance for the study since it was the only class with buried organic layers. On-screen digitizing of ‘shrub wetland’ areas was made using Google Earth’s perspective viewing capability. The land cover classes “steppe forest” and “subalpine forest” showed similar spectral signatures and were separated in ArcGIS 10.5 based on altitudinal criteria defined upon visual interpretation of satellite images and supported by our own field observations. Former “subalpine forest” areas located below 1800 m were reclassified as “steppe forest”. Likewise, former “patchy vegetation” areas located below 1800 m were reclassified as “steppe grassland”. Since we were interested in estimating landscape and land cover SOC stocks in an undisturbed mountain permafrost site, areas in Kuray Basin affected by recent, large-scale human-induced land cover changes, were reclassified according to their former land cover characteristics based on visual interpretation of a 1968 USGS CORONA satellite imagery.

In total, the LCC includes 10 different land cover classes. The accuracy of the LCC was assessed based on 39 ground truth plots combined with 96 manually selected points. All 135 ground truth points were manually classified based on field information and visual interpretations of remotely sensed data. The Kappa index of agreement and Overall accuracy were calculated from a confusion matrix.

The SOC storage of each depth interval for all profiles belonging to the same land cover class was averaged to obtain a mean SOC kg C m^−2^ storage (and its standard deviation) per land cover class. These values were then weighted by the proportion of area covered by each land cover class to estimate the landscape mean SOC storage in the study area. Since no soil profiles were collected for the “mountain forest” class, a SOC default value was assigned to it based on the two lowest elevation profiles in the class “sub-alpine forest”, which had the highest and densest tree canopy. Field observations had shown that the mountain forest area, largely located on moraines, had thin soils with large stone fractions typical of till deposits, similar to those observed at the two sub-alpine forest sites. Land cover units such as perennial snow and water were assumed to contain negligible SOC.

### Statistical analyses

To obtain error estimates for SOC storage in the study area as a whole and its vegetated fraction, 95% confidence interval (CI) ranges were calculated using Eq. (4), where: *t* is the upper α/2 of a normal distribution (*t* = 1.96), *a*_i_ is the total areal extent (%) of the upscaling unit *i*, *SD*_*i*_ is the standard deviation of the upscaling unit *i*, and *n*_*i*_ is the number of replicates in class *i* (Thompson 1992).

4 $${\text{CI}} = t*\sqrt {\sum \left( {\frac{{\left( {a_{i}^{2} *{\text{SD}}_{\text{i}}^{2} } \right)}}{{n_{i} }}} \right)}$$

CI ranges presented here are only an indicator of uncertainties caused by natural variability in the study area and/or by low number of replicates in each class, but do not account for errors in the upscaling products (Hugelius 2012).

## Results

### The land cover classification

**Fig. 3:**
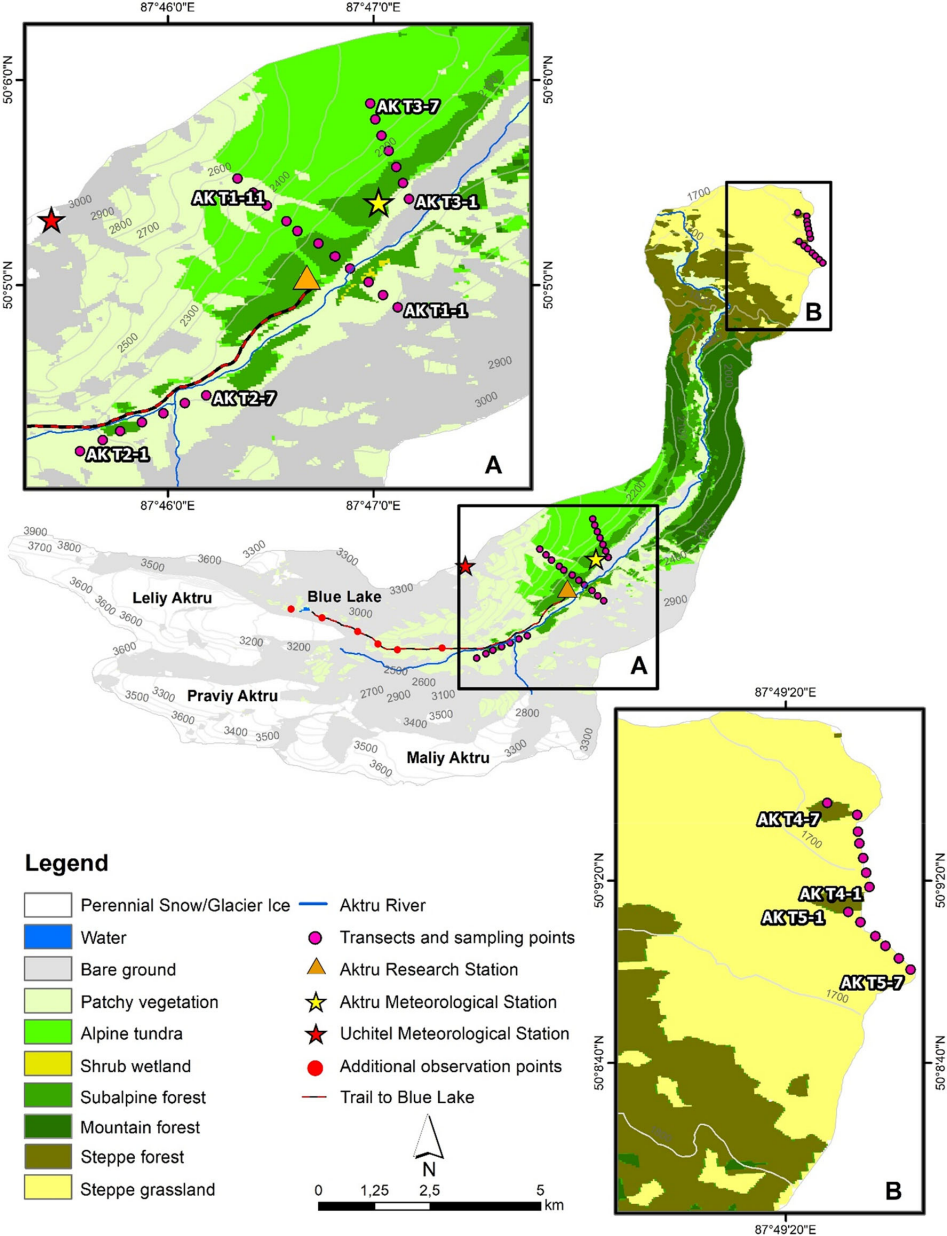
Distribution of land cover classes and locations of interest in the study area, including detailed maps showing transect and sample point locations in Aktru Valley (**a**) and the adjacent portion of Kuray Basin (**b**)

The land cover classification (LCC) presented in Fig. 3 has an overall accuracy of 77% and a Kappa index of agreement of 0.73, similar or higher in comparison with other thematic maps in mountain permafrost settings (e.g., Fuchs et al. 2015). The unvegetated classes occupy 59% and the vegetated classes cover 41% of the study area. Figure 3 shows the location of transects and individual soil profile sites referred to in the text (see also Table S1).

### Radiocarbon dates of selected soil samples

Radiocarbon dates are presented in Table 1. The base of even the thickest topsoil organic layers (17–19 cm deep), encountered in the “subalpine forest” and (by default) “mountain forest” classes (AK T2-4, T3-2 and T3-3), have very recent ages (≤ 220 cal year BP). Oldest dates are recorded for buried organic layers in the “shrub wetland” profile (AK T1-3) and for charred wood in a “subalpine forest” profile (AK T2-3) in the upper reaches of Aktru Valley, but do not exceed 4100 cal year BP. No appropriate materials for dating were found in the lower part of the study area corresponding to Kuray Basin. Topsoil organic layers were thin or absent, and no buried C-enriched materials were encountered in the soil profiles.

**Table 1:** Summary of radiocarbon dating results for buried organics, charred wood, and base topsoil organics, collected in “shrub wetland” (AK 1-3) and “subalpine forest” (AK 2-3, AK 2-4. AK T3-2 and AK T3-3) sites in upper Aktru Valley

Profile code	Depth (cm)	Sample description	Lab. no.^a^	Age ^14^C year BP	Age cal year BP^b^
AK T1-3	45–53	Buried organics	Poz-104130	1140 ± 30	1071
AK T1-3	60–70	Buried organics	Poz-104131	2750 ± 30	2849
AK T2-3	13	Charcoal	Poz-104132	3725 ± 35	4099
AK T2-4	17–18	Base topsoil organics	Poz-104134	230 ± 30	217
AK T3-2	18–19	Base topsoil organics	Poz-104135	102.01 ± 0.33 (pMC^c^)	Modern
AK T3-3	17–18	Base topsoil organics	Poz-104136	103.47 ± 0.37 (pMC^c^)	Modern

^a^Sample number of the Radiocarbon Laboratory in Poznan, Poland

^b^Mean age of 95.4% probability interval expressed in calendar years before 1950

^c^Percent modern carbon

### Typical soil profiles

**Fig. 4:**
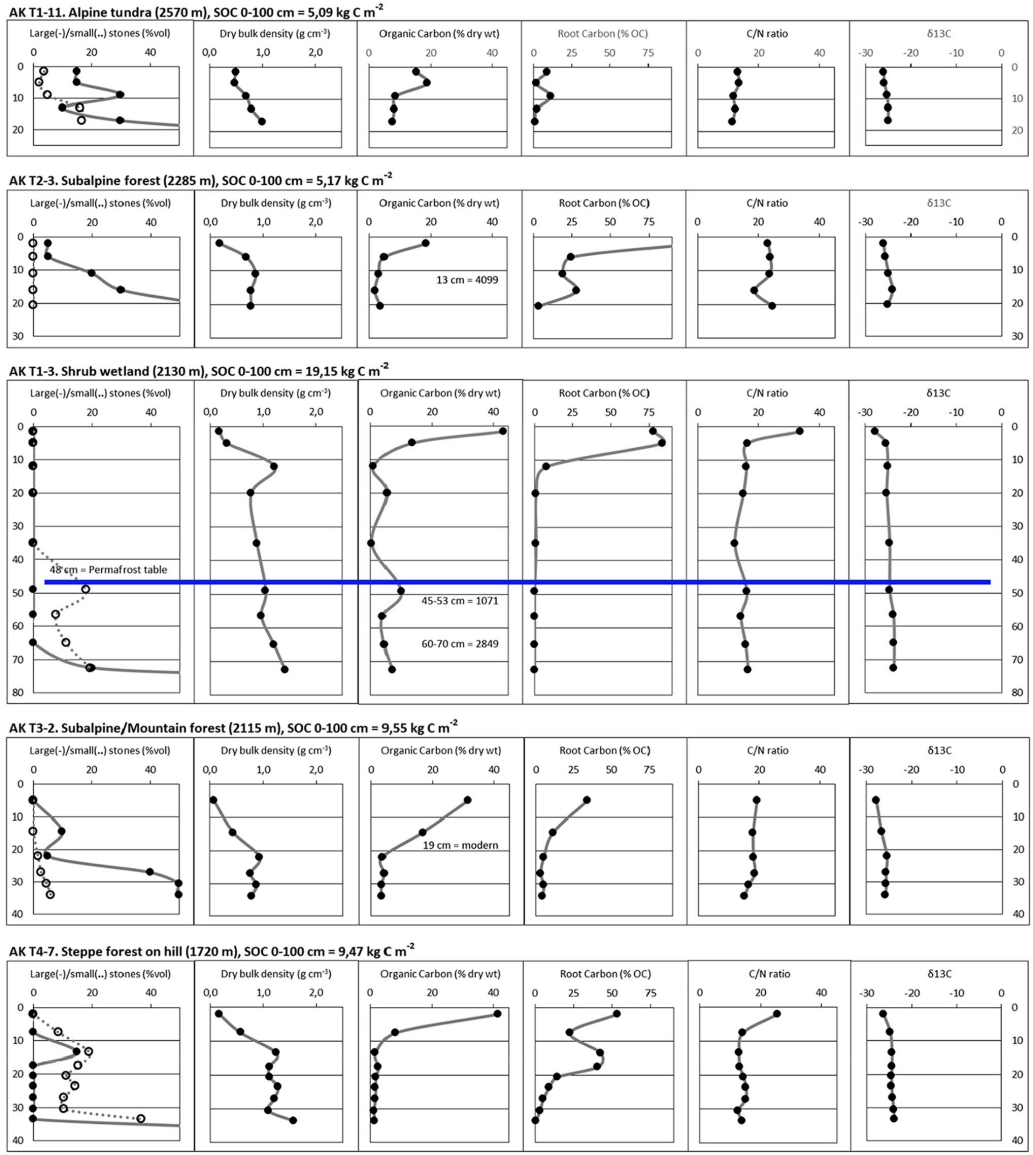

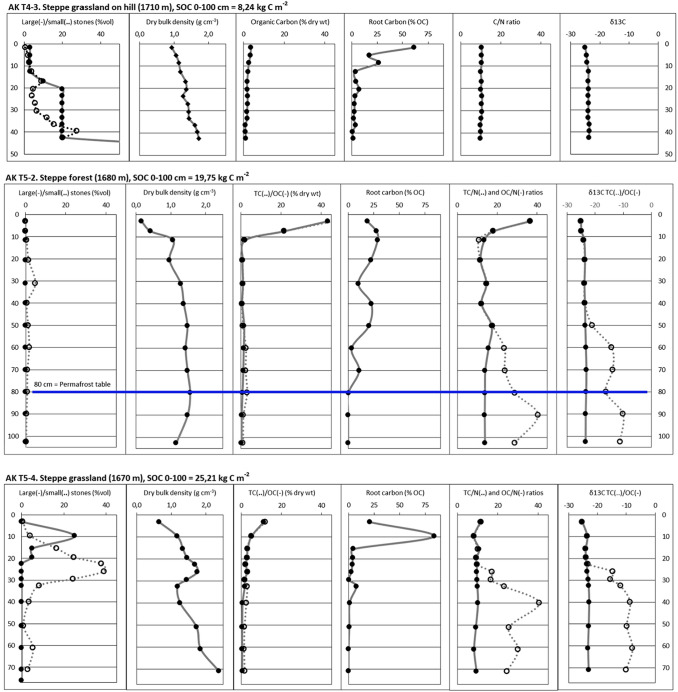
Geochemical properties of selected soil profiles representing the main vegetated land cover classes. Upper permafrost table indicated (where present); The coarse fraction is separated into large stones (> 4 cm diameter) and small stones (< 4 cm diameter); Calibrated radiocarbon ages given in the Organic Carbon column (where available). Soil profiles AK T5-2 and AK T5-4 further differentiate between Total Carbon (TC) and Organic Carbon (OC) %, TC/N and OC/N ratios, and δ^13^C values of TC and OC (to account for Inorganic Carbon)

Figure 4 provides geochemical parameters of selected soil profiles representing the most important vegetated land cover classes in the study area. Soils in the “alpine tundra”, “subalpine forest”, and (by default) “mountain forest” classes of Aktru Valley are quite shallow (AK T1-11, T2-3 and T3-2). In contrast, soils from the Kuray Basin are often thicker (AK T4-3, AK T4-7, AK T5-4), sometimes exceeding 1 m depth (AK T5-2).

In all profiles, the topsoil OL have low DBD, high OC%, root C% and OC/N, and more depleted δ^13^C values, compared to the underlying mineral subsoil layers. Decreasing C/N ratios and less depleted δ^13^C values suggest a higher degree of decomposition of the organic matter with depth.

The “shrub wetland” site in Aktru Valley (AK T1-3) is the only profile in which buried organic layers were encountered (at depths of c. 20, 50 and 70 cm). Radiocarbon ages from 45 to 53 cm (1071 cal year BP) and 60–70 cm (2849 cal year BP) indicate a Late Holocene age for these layers. Geochemical analysis reveals somewhat higher C/N ratios and more depleted δ^13^C values in the buried C-enriched layers compared to the adjacent mineral layers. This suggests slightly less decomposed OM in these buried C-enriched layers, likely due to their quick burial and subsequent preservation under wet anaerobic and permafrost conditions. Permafrost was only reached in this “shrub wetland” site (AK T1-3), at a depth of 48 cm, as well as in a forest steppe site on a slope with northern aspect (AK T5-2), at 80 cm depth. In two soil profiles, the reference depth of 1 m was reached without encountering the upper permafrost table. However, the fact that we did not reach the permafrost layer at a given spot does not necessarily imply that there was no permafrost there.

Significant amounts of IC can be observed in profiles AK T5-2 and AK T5-4, as depicted by anomalously high C/N ratios and less depleted δ^13^C values in bulk samples compared to acid-treated samples. These sites are located on a subdued esker ridge, with material originating from Aktru Valley. Carbonate enrichment can be traced up-valley by C/N and δ^13^C signatures, in profiles AK T3-1 and T1-4 in the glacio-fluvial floodplain deposits and profiles AK T2-7, T2-6, T2-2 and T2-1 in Neoglacial moraine deposits (for location, see Fig. 3). This points to a carbonate bedrock source in upper Aktru Valley.

The percentual contribution of roots to SOC storage in the samples can be high in the upper 10-20 cm of the soil profiles, but generally decreases with depth. The exception is the “forest steppe” site AK T5-2, which has additional root horizons at c. 40 and 70 cm. The latter corresponds to the current active layer depth, the former might represent an older boundary under colder climatic conditions. Larch trees in Siberia are known to have an extensive horizontal rooting system directly above the upper permafrost table (Kajimoto 2010).

### SOC storage upscaling

Mean soil depth weighted by the proportional area representation of each class for the entire study area is 15 cm (Table 2). If we consider only the vegetated area, the weighted mean soil depth is 30 cm. Soils were generally shallow due to the occurrence of stones and bedrock. The reference depth of 1 m was only reached in three out of 39 locations, in the “steppe forest” (*n* = 1) and ‘steppe grassland’ (*n* = 2) land cover classes. Soil depth is a powerful predictor of SOC storage based on all individual soil profiles (*n* = 39; *R*^2^ = 0.81) as well as the means of land cover classes (*n* = 8; *R*^2^ = 0.89).

**Table 2:** Land cover classes, soil profile depths, soil organic carbon (SOC) stocks and root carbon contributions

Land cover classes	Area (%)	Number of sites	Mean ± SD soil profile depth (cm)	Mean ± SD organic layer depth (cm)	Active layer depth (cm)^e^	Mean ± SD SOC 0–100 cm (kg C m^−2^)	Mean ± SD organic layer SOC (kg C m^−2^)	Mean ± SD permafrost (SOC) (kg C m^−2^)	Mean ± SD root C 0–100 cm (kg C m^2^)	Root C contribution to SOC (%)
Perennial snow/glacier ice	25.0	0	0	0		0	0	0	0	0
Water	0.02	0	0	0		0	0	0	0	0
Bare	33.7	3	2.0 ± 3.5	0.0 ± 0.0		0.01 ± 0.01	0	0	0	0
Patchy vegetation	12.6	7	14.1 ± 7.6	0.8 ± 1.9		0.74 ± 1.0	0.4 ± 0.8	0	0.05 ± 0.00 (*n* = 2)	6.6
Alpine tundra	5.3	7	21.0 ± 7.1	5.0 ± 2.7		6.0 ± 5.0	2.5 ± 1.7	0	0.6 ± 0.7 (*n* = 2)	9.5
Shrub wetland	0.02	1	75.0	3.0	48	19.2	1.6	8.5	3.3 (*n* = 1)	17.4
Subalpine forest	3.6	7	28.7 ± 7.9	11.4 ± 6.9		7.8 ± 2.8	5.0 ± 2.0	0	1.7 ± 0.2 (*n* = 5)	22.3
Mountain forest^a^	*6.6*	*(2)*	*32.0 *±* 9.9*	15.5 ± 4.9		*8.1 *±* 2.0*	*6.2 *±* 1.0*	*0*	*1.6 * ± *0.1* (*n* = 2)	*19.4*
Steppe forest	5.7	5	45.0 ± 31.1^c^	4.7 ± 0.7	80 (*n* = 1)^f^	9.5 ± 5.9	3.0 ± 0.6	0.4 ± 0.9	3.0 ± 0.5 (*n* = 2)	31.3
Steppe grassland	7.6	9	47.9 ± 31.7^d^	0.6 ± 1.7		10.3 ± 8.4	0.6 ± 1.8	0	1.9 ± 1.8 (*n* = 2)	18.2
Study area^b^	100	39	13	2.1		2.6 ± 0.6	1.0 ± 0.2	0.03 ± 0.05	0.5 ± 0.2	20.2
Vegetated area^b^	41.4	36	30	5.1		6.2 ± 0.6	2.4 ± 0.2	0.06 ± 0.05	1.2 ± 0.2	20.2

^a^Default values in italic from two subalpine forest sites: AK T1-5 and AK T3-2

^b^Mean weighed SOC storage for study area and vegetated area. with 95% confidence intervals

^c^Full depth of soil profile not reached at one out of five sites (default 100 cm)

^d^Full depth of soil profile not reached at two out of eight sites (default 100 cm)

^e^Upper permafrost table not reached in eight out of 10 land cover classes (permafrost occurrence not confirmed)

^f^Upper permafrost table encountered in only one out of five sites

Weighted mean SOC storage for the top 100 cm of soils in the study area is 2.6 ± 0.6 kg C m^−2^, of which 38% is stored in the topsoil organic layer (1.0 ± 0.1 kg C m^−2^). Excluding unvegetated areas, SOC storage for the 0-100 cm and topsoil organic layer is notably higher with 6.2 ± 0.6 and 2.4 ± 0.2 kg C m^−2^, respectively. Particularly the “subalpine forest” and “mountain forest” classes show high contributions of SOC stored in the topsoil organic layers. The upper permafrost layer (encountered at only two sites) accounts for only c. 1% (0.03 kg C m^−2^) of the total SOC 0–100 cm stored in the study area.

Roots contain 0.5 ± 0.2 kg C m^−2^, which represents c. 20% of the total SOC storage. Highest root stocks are encountered in the “shrub wetland”, all forest and the “steppe grassland” classes. Field observations on deep root penetration in natural exposures showed that only a few fine roots are encountered in the coarse colluvium, till and glacio-fluvial materials underlying the soil profiles, and their contribution to total SOC storage can be considered negligible (< 0.1 kg C m^−2^).

### Landscape partitioning of SOC storage

**Fig. 5:**
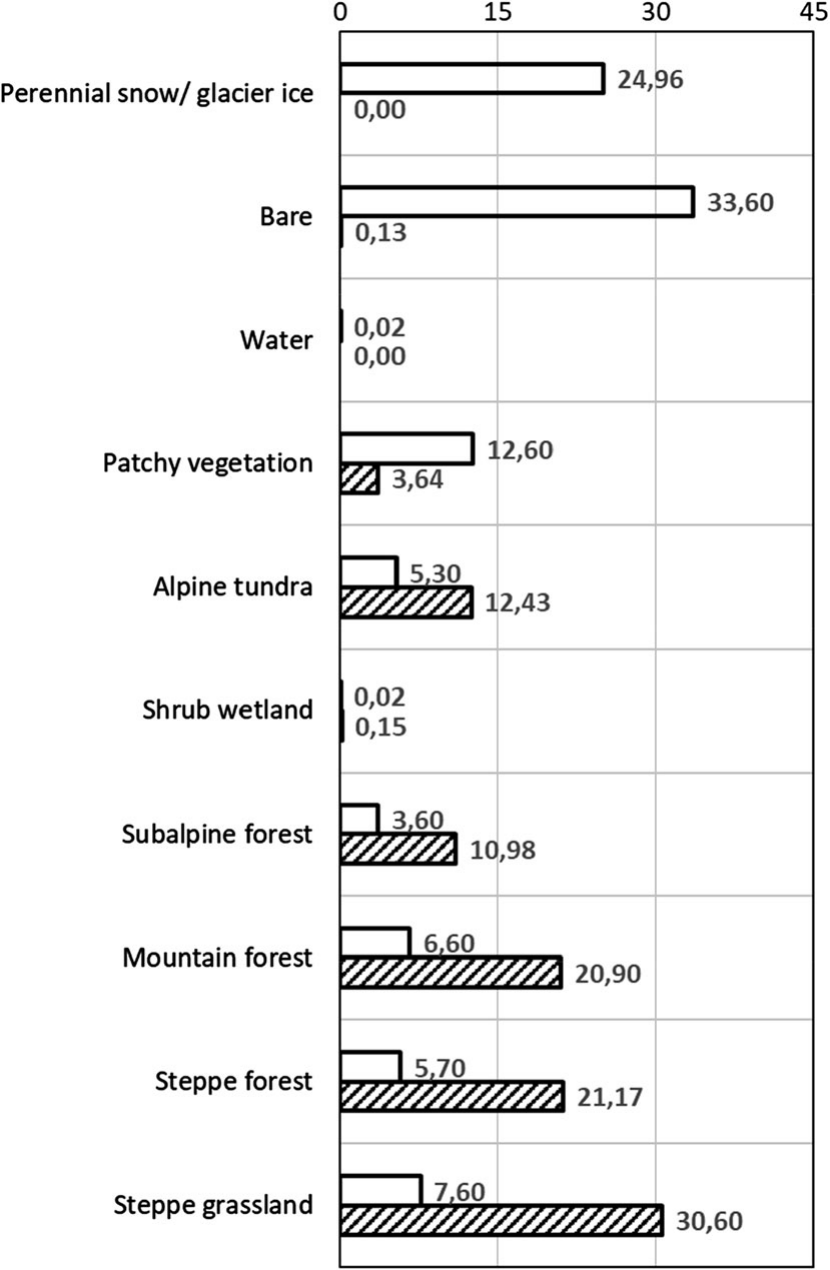
Percent contribution of each land cover class to total study area (white bars) and total SOC 0–100 cm storage (patterned bars)

The high elevation perennial snow/glacier ice and bare ground (including floodplain areas) classes occupy together 59% of the study area, but have negligible SOC stocks (Fig. 5). The high-alpine patchy vegetation (including a minor floodplain area) class accounts for 13% of area but only 3.6% of SOC stocks. The alpine tundra covers 5.3% of the area and holds 12% of the total SOC storage. Forest classes represent 16% of the area and 53% of the SOC stocks. With only 8% of the study area, the “steppe grassland” class represents 31% of the total SOC storage. Among land cover classes, the highest mean SOC 0-100 cm storage is found in the “shrub wetland” class (19.2 kg C m^−2^), but its contribution to total SOC storage in the study area is minimal (0.15%) due to a very low areal coverage (0.02%).

### Topographic controls on SOC storage

**Fig. 6:**
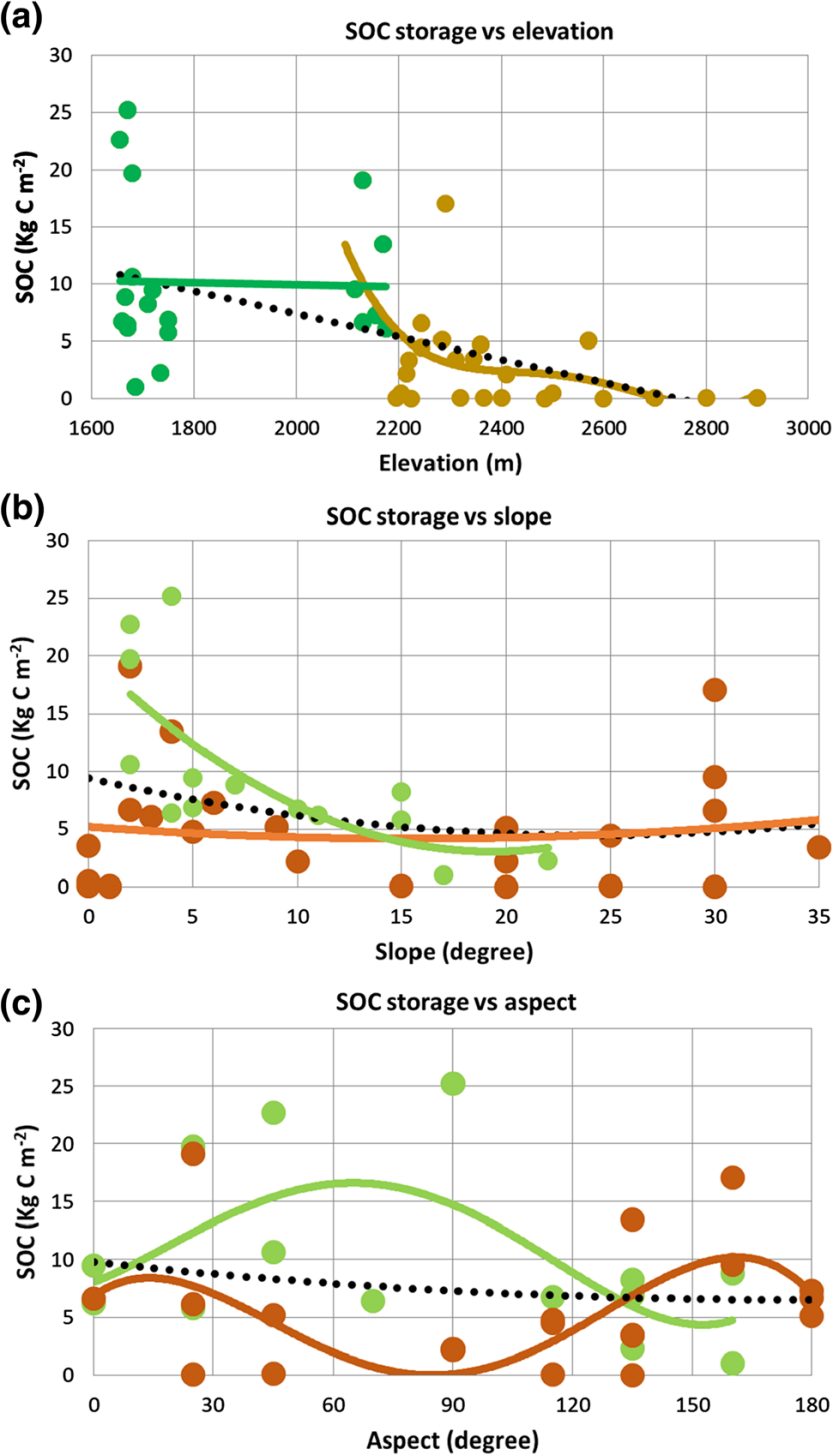
Regressions between SOC 0–100 cm storage and **a** elevation for full data set (dotted black line, *p* < 0.05), soil profiles between 1600 and 2200 m (green line and dots, not significant), and soil profiles between 2100 and 2900 m (brown line and dots, *p* < 0.05); **b** slope within the 0–35° range for full data set (dotted black line, not significant), soil profiles from Aktru Valley (brown line and dots, not significant), and soil profiles from Kuray Basin (green line and dots, *p* < 0.05), and **c** aspect as degree deviation from N for full data set (dotted black line, not significant), soil profiles from Aktru Valley (brown line and dots, *p* < 0.05), and soil profiles from Kuray Basin (green line and dots, *p* < 0.05)

The SOC 0–100 cm stocks at profile sites can be evaluated against their elevation, slope and aspect (Fig. 6a–c). For elevation, we include six additional observation points at approximately 100 m contour intervals between 2400 and 2900 m along the alpine trail between Aktru Research Station (above profile site AK T2-1) and Blue Lake, documenting largely sparsely vegetated and bare areas (Fig. 3). We exclude two bare sites on the active Aktru river floodplain since this type of area can be found in the study area irrespective of elevation. Overall, there is a negative correlation between elevation and SOC storage, but mid- to lower elevation sites between 1600 and 2200 m show no trend. There is a rapid decrease in SOC storage above 2100 m to reach negligible levels above c. 2600 m (Fig. 6a), which can be linked to declining ambient temperatures with elevation.

In terms of slope within the sampled range of 0 to 35°, there are no trends for the study area as a whole or the Aktru Valley. However, there is a significant relationship in the Kuray Basin, with steeper hill slopes (> 15°) holding lower SOC stocks (Fig. 6b). These steeper slopes are correlated with thinner soils as a result of long-term denudation.

When considering all soil profile sites together, there is no statistically significant relationship between aspect and SOC storage (sites with < 2° slope are excluded). However, aspect has different effects in the Kuray Basin compared to the Aktru Valley (Fig. 6c). In the basin area, sites with a NW to E aspect (deviating ≤ 90° from N) have higher SOC stocks compared to those with a more S exposure. This result points to the importance of incoming solar radiation, which affects both surface temperature and humidity. In Aktru Valley, some sites with a NW and SE aspect have high SOC stocks corresponding to stable slopes on the flanks of the E to NE trending valley.

## Discussion

### SOC storage estimates

The estimated mean SOC storage of 2.6 kg C m^−2^ for the 0–100 cm depth interval in Aktru Valley and adjacent Kuray Basin is comparable to those from other permafrost-affected mountain areas, where reported values range between c. 0.5 to 5 kg C m^−2^. Particularly low values of around 1 kg C m^−2^ are reported by Fuchs et al. (2015) for Tarfala Valley (N-Sweden) and by Wojcik et al. (2019) for Brogger Peninsula (Svalbard). These are high mountain and high arctic areas characterized by large proportions of bare grounds without soil development. Higher values of c. 5 kg C m^−2^ are reported by Siewert (2018) for the Abisko area (N Sweden) and by Palmtag et al. (2018) for Zackenberg (NE Greenland). Both these study areas include flat valley bottoms with peat deposits.

At land cover class level, our SOC 0–100 cm estimates for “bare ground” and “patchy vegetation” (0.01 and 0.7 kg C m^−2^, respectively) compare well with the 0.3 and 1.3 kg C m^−2^ yielded in “natural bare lands” and “sparse alpine tundra” in the Urals (NE European Russia) by Kuhry et al. (2002). SOC estimates for “alpine meadow” areas in the Tibetan Plateau yielded values ranging from 3.4 to 10.7 kg C m^−2^ (e.g., Dörfer et al. 2013; Yang et al. 2008), which are similar to “alpine tundra” with 6.0 kg C m^−2^ in Aktru Valley. Zollinger et al. (2013) estimated SOC stocks in “alpine grassland” and “subalpine forest” in the European Alps to be 10 to 15 kg C m^−2^, which are somewhat higher than those found in the “alpine tundra” (6.0 kg C m^−2^) and “subalpine forest” (7.8 kg C m^−2^) in Aktru Valley. Our estimates for “steppe grassland” (10.3 kg C m^2^) and “steppe forest” areas (9.5 kg C m^−2^) are also somewhat lower than the belowground C stocks (down to 1 m) reported by Dulamsuren et al. (2016) for grasslands and Larch forest in the Mongolian Altai (11.6 and 14.0 kg C m^−2^, respectively). Hugelius et al. (2014) reported a much higher mean SOC estimate (16 kg C m^−2^) for the study area in the ‘Northern Circumpolar Soil Carbon Database’ (NCSCDv2 2014), mostly due to an overestimate in the coverage of turbels and orthels, which are very rare or even absent in our SOC inventory.

The mean SOC 0–100 cm estimate for Aktru Valley and the adjacent Kuray Basin is about an order of magnitude lower than in lowland areas across the northern circumpolar permafrost region. For example, Palmtag et al. (2015, 2016) and Siewert et al. (2015) report SOC 0–100 cm storage between 15 and 30 kg C m^−2^ for lowland tundra and forest areas in central and northern Siberia. Furthermore, between 23 and 47% of the SOC 0–100 cm stock in these lowland areas is stored in the permafrost layer. Our much lower storage can be explained by multiple factors. About 59% of the study area is covered by glaciers and bare ground in upper Aktru Valley, holding negligible SOC. Soils in the vegetated areas have large amounts of stones both on the surface (inhibiting vegetation development) and within the profile (decreasing the fraction of soil volume containing SOC). Vegetation development and soil formation is also hindered by steep topography, resulting in thin soils. Coarse sediments resulting from glacial activity and active slope processes enhance water drainage, which in turn favors aerobic SOM decomposition and inhibits cryoturbation. As a result, SOC in this type of mountain permafrost soils is often concentrated in the top 30 cm of the profile (Bockheim and Munroe 2014). The permafrost table generally occurs at greater depths than the active soil formation, implying that there is almost no SOC prevented from decomposition by sub-zero temperatures. In our study, SOC stored in permafrost accounted for only 1% of the total stored SOC. Soils with buried organic layers in both the active layer and the upper permafrost layer were only described from the “shrub wetland” class, which is restricted to a very small proportion (0.02%) of the total study area.

### Global warming impacts on SOC storage

Among land cover classes, the more densely vegetated surfaces (the ‘alpine tundra’, all forest and ‘steppe grassland’ classes), covering 29% of the study area, account for 96% of total SOC storage. Our results point to limited differences in total SOC storage between land cover classes in the middle-lower Aktru Valley and the adjacent intra-montane Kuray Basin. The different forest classes have a narrow range in SOC 0-100 cm values between 7.8 to 9.5 kg C m^−2^, and steppe grassland has only a slightly higher value of 10.3 kg C m^−2^. Our topographic analyses show no relationship with the elevation (temperature) gradient, with slope and aspect governing SOC storage in Kuray Basin. Any future changes in effective precipitation might affect the proportional representation of these different land cover classes, but we anticipate only small changes in total SOC allocation in this part of the study area.

SOC storage in the upper part of Aktru Valley is strongly controlled by the elevation (temperature) gradient, outside of areas with steep bedrock (> 35º), active talus slopes and active floodplains. Mean SOC 0–100 cm values increase from 7.8 kg C m^−2^ in subalpine forest below treeline (c. 2300 m), through 6.0 kg C m^−2^ in alpine tundra (up to 2600 m), to < 1 kg C m^−2^ in sparsely vegetated and bare areas at higher elevations. According to IPCC (2013), mean annual air temperature increases in the order of 3–5 °C are projected for Central Asia by the end of this Century. Applying the current altitudinal thermal lapse rate of 0.58 °C/100 m for the month of July (growing season), this would imply an altitudinal shift in plant life zones of c. 500–900 m. Since the amount of SOC currently found in the permafrost layer of soils is minimal, only negligible amounts of SOC will be exposed to permafrost degradation and microbial decomposition under future global warming. Potentially, large proportions of currently bare areas at high elevations in Aktru Valley would become suitable for plant growth, litter input and soil development resulting in increased phytomass C uptake and SOC storage. This increase in C uptake can only be hindered in steep areas where permafrost degradation would cause an increase in slope instability (Gregory and Geoudie 2011).

An upward altitudinal shift of vegetation zones and glacier retreat has already been observed in upper Aktru Valley due to a rapid warming in recent decades (Davydov and Timoshok 2010; Nazarov et al. 2016; Gatti et al. 2019). Davydov and Timoshok (2010) estimated the root biomass in the top 10 cm of soils after 50 and 100 years of glacier retreat to be 0.25 and 0.75 kg C m^−2^, respectively. In our study, sites deglaciated since the Little Ice Age (< 150 years old) yielded total SOC values ranging from 0.1 to 2 kg C m^−2^. Furthermore, ‘modern’ basal radiocarbon dates in thick topsoil organic layers accounting for c. 7 kg C m^−2^ storage at two of our subalpine forest sites indicate rapid potential SOC accretion under favorable environmental conditions. This suggests that, in contrast to lowland permafrost areas in the northern circumpolar permafrost region, an alpine permafrost area such as upper Aktru Valley has to be considered a C sink in the future, implying a negative feedback on global warming. A careful spatial analysis of the global permafrost area is still pending, but it is most likely that the relatively small C uptake in bare mountain and high arctic settings will be outweighed by the much larger C losses resulting from thawing and subsequent decomposition of the large SOC pool in lowland regions (Schuur et al. 2009), resulting in a net positive permafrost C feedback on global warming.

## Conclusions

This study provides new SOC data for the Russian High Altai region. The mean SOC storage for the upper 100 cm of soils in Aktru Valley and the adjacent Kuray Basin (2.6 kg C m^−2^) is about an order of magnitude lower than in any lowland permafrost area, and the permafrost layer in our study area stores negligible SOC (0.03 kg C m^−2^).

Our data indicate that a mountain permafrost environment such as Aktru Valley cannot be considered an important C source under future global warming and permafrost thawing. Instead, it might become a C sink due to an upward shift of plant life zones resulting in increased phytomass C and SOC storage.

In addition, our SOC storage estimate is 6 to 7 times lower than that reported in the ‘Northern Circumpolar Soil Carbon Database’ (NCSCDv2 2014) for our study area. This study emphasizes the need for more quantitative SOC estimates in mountain permafrost areas to conduct a comprehensive global assessments of the permafrost C feedback on climate change.

## Electronic supplementary material

Below is the link to the electronic supplementary material.

Supplementary material 1 (PDF 228 kb)

## References

[CR1] Agatova AR, Nazarov AN, Nepop RK, Rodnight H (2012). Holocene glacier fluctuations and climate changes in the southeastern part of the Russian Altai (South Siberia) based on a radiocarbon chronology. Quaternary Science Reviews.

[CR2] Bockheim JG, Munroe JS (2014). Organic carbon pools and genesis of alpine soils with permafrost: A review. Arctic, Antarctic, and Alpine Research.

[CR3] Bronk Ramsey, C. 2019. OxCal 4.3. Retrieved June 10, 2019, from https://c14.arch.ox.ac.uk/oxcal/OxCal.html.

[CR4] Callaghan, T.V., O.M. Shaduyko, and S.N. Kirpotin. 2021. Siberian Environmental Change. Special Issue. *Ambio*. Volume 50. 10.1007/s13280-021-01626-7 PMC8479719 34586591

[CR5] Campbell J (2011). Introduction to remote sensing.

[CR6] Davydov V, Timoshok E (2010). Forming of soils on young moraines in the basin of the Aktru Glacier (Central Altai, North-Chuya Ridge). Contemporary Problems of Ecology.

[CR7] Dörfer C, Kühn P, Baumann F, He JS, Scholten T (2013). Soil organic carbon pools and stocks in Permafrost-Affected soils on the Tibetan Plateau. PLoS ONE.

[CR8] Dulamsuren C, Klinge M, Degener J, Khishigjargal M, Chenlemug T, Bat-enerel B, Yeruult Y, Saindovdo D (2016). Carbon pool densities and a first estimate of the total carbon pool in the Mongolian forest-steppe. Global Change Biology.

[CR9] Fuchs M, Kuhry P, Hugelius G (2015). Low below-ground organic carbon storage in a subarctic Alpine permafrost environment. Cryosphere.

[CR10] Galakhov, V., Y. Narozhnev, and S. Nikitin. 1987. The Aktru Glaciers (Altai). *Gidrometeoizdat*, 119 pp. (in Russian).

[CR11] Gatti RC, Callaghan TV, Velichevskaya A, Dudko A, Fabbio L, Battippaglia G, Liang J (2019). Accelerating upward treeline shift in the Altai Mountains under last-century climate change. Nature Science Reports. Scientific Reports.

[CR12] Gregory KJ, Goudie SA (2011). The SAGE handbook of geomorphology.

[CR13] Gruber N, Friedlingstein P, Field CB, Valentini R, Heimann M, Richey JE, Romero-Lankao P, Schulze D, Field CB, Raupach M (2004). The vulnerability of the carbon cycle in the 21st century: an assessment of carbon-climate-human interactions. The global carbon cycle, integrating humans, climate and the natural world.

[CR14] Hugelius G (2012). Spatial upscaling using thematic maps: An analysis of uncertainties in permafrost soil carbon estimates. Global Biogeochemical Cycles.

[CR15] Hugelius G, Strauss J, Zubrzycki S, Harden JW, Schuur EAG, Ping C-L, Schirrmeister L, Grosse G (2014). Estimated stocks of circumpolar permafrost carbon with quantified uncertainty ranges and identified data gaps. Biogeosciences.

[CR16] IPCC, 2013. Climate Change 2013: The physical science basis. Contribution of Working Group I to the Fifth Assessment Report of the Intergovernmental Panel on Climate Change. In ed. T.F. Stocker, D. Qin, G.-K. Plattner, M. Tignor, S.K. Allen, J. Boschung, A. Nauels, Y. Xia, V. Bex and P.M. Midgley. Cambridge: Cambridge University Press, 1535 pp.

[CR17] Kajimoto T, Osawa A, Zyryanova OA, Matsuura Y, Kajimoto T, Wein RW (2010). Root system development of Larch trees growing on siberian permafrost. Permafrost ecosystems, Siberian larch forests.

[CR18] Kuhry P, Vitt D (1996). Fossil carbon/nitrogen ratios as a measure of peat decomposition. Ecology.

[CR19] Kuhry P, Mazhitova GG, Forest P-A, Deneva SV, Virtanen T, Kultti S (2002). Upscaling soil organic carbon estimates for the Usa Basin (Northeast European Russia) using GIS-based landcover and soil classification schemes. Geografisk Tidsskrift-Danish Journal of Geography.

[CR20] Lehmkuhl F, Klinge M, Stauch G, Ehlers J, Gibbards PL (2011). The extent and timing of Late Pleistocene Glaciations in the Altai and neighboring mountains systems. Development in quaternary science—extent and chronology—a closer look.

[CR21] Li Y, Zhang D, Andreeva M, Li Y, Fan L, Tang M (2020). Temporal-spatial variability of modern climate in the Altai Mountains during 1970–2015. PLoS ONE.

[CR22] Narozhniy Y, Zemtsov V (2011). Current state of the Altai Glaciers (Russia) and trends over the period of instrumental observations 1952–2008. Ambio.

[CR23] Nazarov AN, Myglan V, Orlova LA, Ovchinnikov IY (2016). Activity of Maly Aktru Glacier (Central Altai) and changes tree line fluctuations in its basin for a historical period. Ice and Snow.

[CR24] NCSCD version 2. 2014. 10.5879/ecds/00000002.

[CR25] Palmtag J, Cable S, Christiansen HH, Hugelius G, Kuhry P (2018). Landscape partitioning and estimates of deep storage of soil organic carbon in the Zackenberg area (NE Greenland) using geomorphological landforms. The Cryosphere.

[CR26] Palmtag J, Hugelius G, Lashchinskiy N, Tamstorf MP, Richter A, Elberling B, Kuhry P (2015). Storage, landscape distribution, and burial history of soil organic matter in contrasting areas of continuous Permafrost. Arctic, Antarctic, and Alpine Research.

[CR27] Palmtag J, Ramage J, Hugelius G, Gentsch N, Lashchinskiy N, Richter A, Kuhry P (2016). Controls on the storage of organic carbon in permafrost soil in northern Siberia. European Journal of Soil Science.

[CR28] Ping CL, Jastrow JD, Jorgenson MT, Michaelson GJ, Shur YL (2015). Permafrost soils and carbon cycling. Soil.

[CR29] Rytter R (2012). Stone and gravel contents of arable soils influence estimates of C and N stocks. CATENA.

[CR30] Schuur E, McGuire AD, Schädel C, Grosse G, Harden JW, Hayes DJ, Hugelius G, Koven CD (2015). Climate change and the permafrost carbon feedback. Nature.

[CR31] Schuur E, Vogel JG, Crummer KG, Lee H, Sickman JO, Osterkamp TE (2009). The effect of permafrost thaw on old carbon release and net carbon exchange from tundra. Nature.

[CR32] Sevastyanov VV (1998). Climate of alpine areas of the Altai and Sayan mountains.

[CR33] Sevastyanova IM, Sevastyanov VV (2013). Climatic resources of the aktru mountainous-glacial representative basin. Altai.

[CR34] Siewert MB (2018). High-resolution digital mapping of soil organic carbon in permafrost terrain using machine learning: a case study in a sub-Arctic peatland environment. Biogeosciences.

[CR35] Siewert MB, Hanisch J, Weiss N, Kuhry P, Maximov TC, Hugelius G (2015). Comparing carbon storage of Siberian tundra and taiga permafrost ecosystems at very high spatial resolution. Journal of Geophysical Research -Biogeosciences.

[CR36] Tarnocai C, Canadell JG, Schuur EAG, Kuhry P, Mazhitova G, Zimov S (2009). Soil organic carbon stocks in the northern circumpolar permafrost region. Global Biogeochemical Cycles.

[CR37] Thompson S (1992). Sampling.

[CR38] Tronov M, Olejnik I, Shantykova L (1973). Experience of complex research of water balance in mountainous-glacial representative basin (Aktru basin in Altai). Glacio-hydro-climatology of highlands.

[CR39] UNFCCC, 2015. Adoption of the Paris agreement. COP21, Paris.

[CR40] Wojcik R, Palmtag J, Hugelius G, Weiss N, Kuhry P (2019). Land cover and landform-based upscaling of soil organic carbon stocks on the Brogger Peninsula, Svalbard. Arctic, Antarctic, and Alpine Research.

[CR41] World Data Center (RIHMI-WDC). Retrieved 14 March, 2018. https://www.bodc.ac.uk/resources/inventories/edmed/org/681/.

[CR42] Yang Y, Fang J, Tang Y, Ji C, Zheng C, He J, Zhu B (2008). Storage, patterns and controls of soil organic carbon in the Tibetan grasslands. Global Change Biology.

[CR43] Yershov, E. D. 2003. (ed). Geocryological map of Russia and neighbouring republics (2nd Edition), Map Sheet 14. English language version, ed. P.J. Williams, and M.T. Warren. Collaborative map project, Ottawa, Canada, 20 p.

[CR44] Zhang D, Yang Y, Lan B (2018). Climate variability in the northern and southern Altai Mountains during the past 50 years. Scientific Reports.

[CR45] Zollinger B, Alewell C, Kneisel C, Meusburger K, Gärtner H, Brandová D, Ivy-Ochs S, Schmidt MWI (2013). Effect of permafrost on the formation of soil organic carbon pools and their physical-chemical properties in the Eastern Swiss Alps. CATENA.

