# Supplementary material for: Soil organic carbon storage in a mountain permafrost area of Central Asia (High Altai, Russia)
Source: Ambio. 2020 Dec 7;50(11):2022–37. doi: 10.1007/s13280-020-01433-6 (PMC8497679; doi:10.1007/s13280-020-01433-6)
Supplement: Supplementary file 1 — Supplementary material 1 (PDF 228 kb) [file 13280_2020_1433_MOESM1_ESM.pdf]

**Ambio**

Electronic Supplementary Material

This supplementary material has not been peer reviewed

Title: **Soil organic carbon storage in a mountain permafrost area of Central Asia (High Altai, Russia)**

Table S1. Transect and profile codes, geographic coordinates, topographic parameters, soil profile depth, land cover class, mean height of upper vegetation stratum, and percent cover of plant functional types and mineral grounds.

| Transect/ Profile | Latitude N (degrees, minutes) | Longitude E (degrees, minutes) | Elevation (m) | Slope (degrees) | Aspect (degrees) | Profile Depth (cm) | Land Cover Class | Height Vegetation [c] (m) | Deciduous coniferous Larch (%) | Evergreen coniferous Pine (%) | Deciduous Shrub (%) | Evergreen Dwarf Shrub (%) | Graminoid (%) | Forb (%) | Moss (%) | Lichen (%) | Litter (%) | Bare Ground (%) | Large Stones (%) |
|---|---|---|---|---|---|---|---|---|---|---|---|---|---|---|---|---|---|---|---|
| | | | | | | | | | | | | | | | | | | | |
| AK T1-1 | 50 04.891 | 87 47.119 | 2225 | 30 | 335 | 0 | Bare | 0,30 | | | | | | <1 | | | | 29 | 70 |
| AK T1-2 | 50 04.951 | 87 47.046 | 2175 | 3 | 335 | 21 | Subalpine forest | 8,00 | 20 | 1 | | 70 | 2 | | 25 | 25 | 20 | 0 | 1 |
| AK T1-3 | 50 05.015 | 87 46.976 | 2130 | 2 | 335 | 75 | Shrub wetland | 1,00 | | | 65 | | 40 | 10 | 60 | | | 0 | 0 |
| AK T1-4 | 50 05.080 | 87 46.886 | 2115 | 1 | 315 | 12 | Patchy vegetation | 0,05 | | | | | 3 | 2 | | | | 45 | 50 |
| AK T1-5 | 50 05.140 | 87 46.814 | 2130 | 2 | 180 | 25 | Subalp/Mtn forest [b] | 12,0 | 10 | 50 | | 20 | 3 | 15 | 25 | 20 | | 0 | 0 |
| AK T1-6 | 50 05.203 | 87 46.735 | 2170 | 4 | 135 | 26 | Subalpine forest | 3,00 | | 10 | 10 | 2 | 20 | 20 | | | 40 | 0 | 0 |
| AK T1-7 | 50 05.263 | 87 46.632 | 2245 | 25 | 115 | 20 | Alpine tundra | 7,00 | 10 | | | 1 | 40 | 30 | | | 15 | 5 | 0 |
| AK T1-8 | 50 05.310 | 87 46.578 | 2310 | 35 | 135 | 21 | Alpine tundra | 0,20 | | | 5 | 3 | 20 | 5 | | 1 | 20 | 10 | 10 |
| AK T1-9 | 50 05.387 | 87 46.482 | 2410 | 20 | 90 | 29 | Patchy vegetation | 0,25 | | | 5 | | 3 | 1 | | | 3 | 30 | 60 |
| AK T1-10 | 50 05.450 | 87 46.416 | 2485 | 20 | 135 | 0 | Bare | 0,20 | | | <1 | <1 | | | <1 | 10 | | 0 | 99 |
| AK T1-11 | 50 05.519 | 87 46.339 | 2570 | 20 | 180 | 23 | Alpine tundra | 0,05 | | | | 25 | 10 | 5 | 1 | 2 | 20 | 1 | 15 |
| | | | | | | | | | | | | | | | | | | | |
| AK T2-1 | 50 04.190 | 87 45.572 | 2365 | 0 | none | 8 | Patchy vegetation | 0,20 | | | 3 | 4 | | | 1 | | 3 | 55 | 35 |
| AK T2-2 | 50 04.244 | 87 45.681 | 2320 | 25 | 45 | 19 | Patchy vegetation | 3,50 | | 10 | | 5 | | | | 1 | 5 | 25 | 65 |
| AK T2-3 | 50 04.288 | 87 45.767 | 2285 | 9 | 45 | 29 | Subalpine forest | 7,00 | | 20 | 30 | 2 | 5 | | 10 | 50 | 10 | 0 | 5 |
| AK T2-4 | 50 04.331 | 87 45.873 | 2245 | 30 | 360 | 40 | Subalpine forest | 1,50 | 3 | 3 | 50 | 3 | 2 | 1 | 50 | | 20 | 0 | 0 |
| AK T2-5 | 50 04.375 | 87 45.977 | 2215 | 10 | 90 | 8 | Patchy vegetation | 3,00 | | 10 | 70 | 1 | | 1 | 1 | 1 | 90 | 0 | 10 |
| AK T2-6 | 50 04.425 | 87 46.082 | 2205 | 0 | none | 13 | Patchy vegetation | 6,00 | 20 | 5 | 3 | 15 | | 5 | 3 | 3 | 50 | 5 | 30 |
| AK T2-7 | 50 04.463 | 87 46.187 | 2195 | 15 | 115 | 10 | Patchy vegetation | 0,10 | | | 5 | | | 3 | 1 | | 1 | 30 | 65 |
| | | | | | | | | | | | | | | | | | | | |
| AK T3-1 | 50 05.420 | 87 47.173 | 2095 | 1 | 45 | 6 | Bare | 0,05 | | | | | | <1 | 1 | | | 49 | 50 |
| AK T3-2 | 50 05.497 | 87 47.143 | 2115 | 30 | 160 | 39 | Subalp/Mtn forest [b] | 13,0 | 40 | 10 | 1 | 5 | 75 | 25 | | | 3 | 0 | 0 |
| AK T3-3 | 50 05.575 | 87 47.111 | 2155 | 6 | 180 | 21 | Subalpine forest | 10,0 | 40 | 2 | 25 | | 50 | 15 | | | 5 | 0 | 0 |
| AK T3-4 | 50 05.654 | 87 47.074 | 2220 | 35 | 135 | 18 | Alpine tundra | 0,70 | | | | | 40 | 50 | | | 20 | 0 | 0 |
| AK T3-5 | 50 05.729 | 87 47.039 | 2290 | 30 | 160 | 35 | Alpine tundra | 0,70 | | | 10 | | 30 | 30 | | | 20 | 0 | 0 |
| AK T3-6 | 50 05.808 | 87 47.008 | 2345 | 0 | none | 12 | Alpine tundra | 0,20 | | | 5 | 20 | 30 | 5 | | 1 | 10 | 10 | 30 |
| AK T3-7 | 50 05.887 | 87 46.982 | 2360 | 5 | 115 | 18 | Alpine tundra | 0,30 | | | 80 | 1 | 10 | 1 | | 2 | 10 | 0 | 0 |
| | | | | | | | | | | | | | | | | | | | |
| AK T4-1 | 50 09.310 | 87 49.640 | 1670 | 4 | 70 | 32 | Steppe forest | 12,0 | 50 | | | | 60 | 20 | | | 20 | 0 | 0 |
| AK T4-2 | 50 09.362 | 87 49.628 | 1685 | 17 | 160 | 12 | Steppe grassland | 0,20 | | | 15 | | 50 | 15 | | 2 | 5 | 15 | 15 |
| AK T4-3 | 50 09.416 | 87 49.617 | 1710 | 15 | 135 | 48 | Steppe grassland | 0,25 | | | 2 | | 60 | 1 | | 1 | 3 | 30 | 3 |
| AK T4-4 | 50 09.469 | 87 49.604 | 1735 | 22 | 135 | 28 | Steppe grassland | 0,25 | | | | 5 | 50 | 2 | | 3 | 5 | 15 | 30 |
| AK T4-5 | 50 09.512 | 87 49.598 | 1750 | 5 | 135 | 33 | Steppe grassland | 0,25 | | | | | 80 | 15 | | 3 | 2 | 0 | 5 |
| AK T4-6 | 50 09.573 | 87 49.595 | 1750 | 15 | 335 | 26 | Steppe forest | 10,0 | 60 | | 5 | 5 | 10 | 1 | 60 | 1 | 20 | 0 | 0 |
| AK T4-7 | 50 09.615 | 87 49.487 | 1720 | 5 | 360 | 38 | Steppe forest | 8,00 | 20 | | 25 | | 15 | 3 | 40 | | 25 | 0 | 0 |
| | | | | | | | | | | | | | | | | | | | |
| AK T5-1 | 50 09.270 | 87 49.504 | 1670 | 11 | 360 | 29 | Steppe forest | 12,0 | 30 | | | 1 | 50 | 15 | 40 | | 30 | 0 | 0 |
| AK T5-2 | 50 09.219 | 87 49.563 | 1680 | 2 | 335 | >110 [a] | Steppe forest | 10,0 | 30 | | 10 | 10 | 30 | 5 | 75 | 1 | 10 | 0 | 0 |
| AK T5-3 | 50 09.181 | 87 49.607 | 1680 | 2 | 315 | 24 | Steppe grassland | 7,00 | 10 | | 5 | | 75 | 10 | | | 10 | 0 | 0 |
| AK T5-4 | 50 09.130 | 87 49.662 | 1670 | 4 | 90 | >76 [a] | Steppe grassland | 0,15 | | | | 3 | 70 | 10 | | | 20 | 2 | 0 |
| AK T5-5 | 50 09.094 | 87 49.699 | 1665 | 7 | 160 | 50 | Steppe grassland | 0,20 | | | | | 50 | 5 | | 2 | 20 | 20 | 15 |
| AK T5-6 | 50 09.049 | 87 49.748 | 1660 | 10 | 115 | 36 | Steppe grassland | 0,15 | | | | 5 | 40 | 3 | | 3 | 3 | 40 | 10 |
| AK T5-7 | 50 09.008 | 87 49.789 | 1655 | 2 | 315 | >96 [a] | Steppe grassland | 0,50 | | | 10 | | 70 | 5 | | | 10 | 0 | 0 |

[a] Maximum soil depth not reached at three sites

[b] Subalpine forest sites used as default sites for the Mountain forest class

[c] Value indicates mean height of the upper vegetation stratum (even when sparse)
